# Synchronous and metachronous malignant melanomas arising in a human immunodeficiency virus-positive patient after the commencement of highly active antiretroviral therapy treatment: a case report

**DOI:** 10.1186/s13256-021-02920-4

**Published:** 2021-07-14

**Authors:** Sharad P. Paul, Simon Briggs, Michael Hitchcock

**Affiliations:** 1 Faculty of Surgery, University of Auckland, Auckland, New Zealand; 2 Auckland District Health Board, Auckland, New Zealand

**Keywords:** Melanoma, HIV, Antiretroviral drugs, Skin cancer

## Abstract

**Background:**

We present an unusual case of a patient who developed four melanomas within a few months of diagnosis with human immunodeficiency virus and commencement of highly active antiretroviral therapy therapy. The patient had no previous history of melanoma, and previous skin checks were normal.

**Case presentation:**

A 50-year-old Caucasian male drainlayer with Fitzpatrick type 2 skin presented for a routine skin examination. He had been diagnosed with human immunodeficiency virus 4 months earlier and commenced on highly active antiretroviral therapy therapy. He was found to have three melanomas (melanoma *in situ* stage) on excision biopsies, and when he presented for wider excisions of these sites a few weeks later, another new melanoma *in situ* was found. He had no other medical history of note, and no symptoms to report. He is being followed up 3-monthly.

**Conclusions:**

This case of a human immunodeficiency virus-positive person presenting with four cutaneous melanomas—occurring in both synchronous and metachronous fashion within a 4-month period—is being presented both for its uniqueness and also to highlight the increased need for close skin surveillance in human immunodeficiency virus-positive patients.

## Introduction

Human immunodeficiency virus (HIV) infection has been associated with skin manifestations of cancers such as Kaposi’s sarcoma and non-Hodgkin’s lymphoma. Even in the post-highly active antiretroviral therapy (HAART) era, the risk of melanoma in those with HIV/acquired immunodeficiency syndrome (AIDS) remains elevated. We present an unusual case of a patient who developed four melanomas within a few months of diagnosis of HIV and commencement of HAART therapy.

## Case presentation

Demographic details: 50-year-old Caucasian male drainlayer with Fitzpatrick type 2 skin

Medical history: Recent diagnosis of HIV (4 months ago) and commencement of HAART therapy (2 months ago). No other medical conditions.

Symptoms and signs: Nil. Patient was not aware of any skin issues and presented for a routine skin examination given his outdoor occupation. Three pigmented lesions suspicious for melanoma were identified on his trunk, which turned out to be melanoma *in situ* (MIS).

Treatment or intervention and outcomes: following wide excision of the MIS sites, the patient is well and is being followed up 3-monthly with skin examinations. Another MIS and a nodular basal cell carcinoma (BCC) have since been diagnosed, and excised.

## Clinical history

As summarized above, a 50-year-old Caucasian male drainlayer presented for a routine skin examination given his outdoor occupation and the approaching summer season. Of note, he had been diagnosed with HIV 4 months earlier, and commenced on HAART therapy with dolutegravir, emtricitabine, and tenofovir 2 months prior to this skin examination. He had no other medical conditions or symptoms, and his past history was unremarkable. He was continuing to work actively as a drainlayer and was not aware of any suspicious lesions on his skin. He had Fitzpatrick type 2 skin, but no previous history of skin cancer. Three small 2–3 mm pigmented lesions on his trunk were suspicious for melanoma (left shoulder, lower back, and right shoulder) and excision biopsies were performed on these lesions. Histology confirmed these lesions to be melanoma *in situ* (MIS), and the patient was booked for wider excisions to ensure 5-mm margins in keeping with melanoma guidelines.

**Fig. 1:**
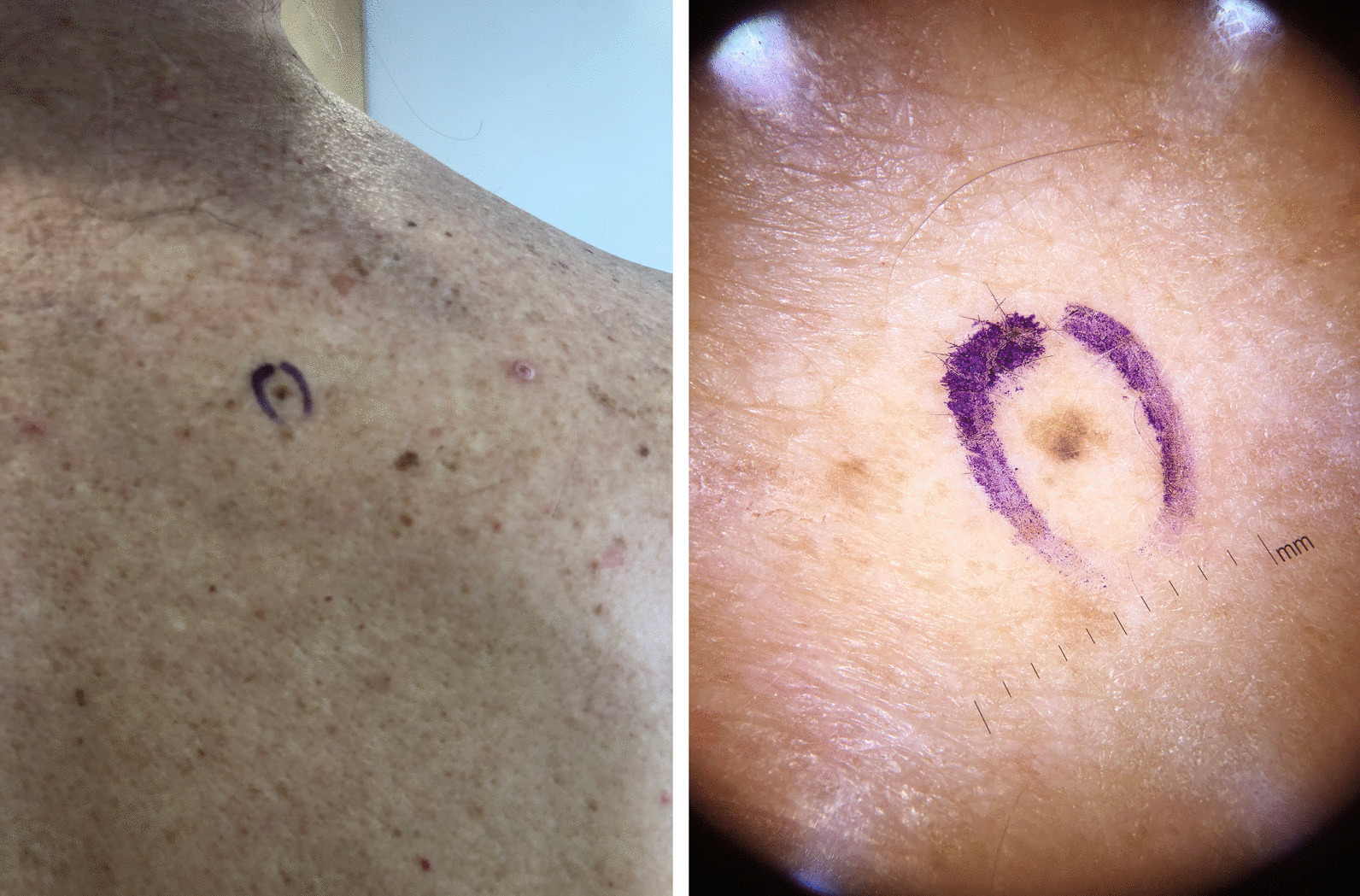
Clinical and dermatoscopic photographs of the pigmented lesion (MIS) noted on the right scapular region

During these wider excisions, which were done approximately 8 weeks after his initial excisions, a new pigmented lesion on his right scapular region had appeared, which also appeared atypical on dermatoscopy; this lesion turned out to be another MIS—his fourth in 4 months. Dermatoscopy of these MIS lesions, given their very recent, sudden onset and small size—around 2 mm—showed mainly irregular hyperpigmented areas (Fig. 1).

This case of a HIV-positive person presenting with four cutaneous melanomas—occurring in both synchronous and metachronous fashion within a 4-month period—is being presented both for its uniqueness and to highlight the increased need for close skin surveillance in HIV-positive patients.

## Investigations

At the time of his HIV diagnosis, his CD4 count was 83 cells/µL (5%). This is a low CD4 count (anything below 200 is considered low).

**Fig. 2:**
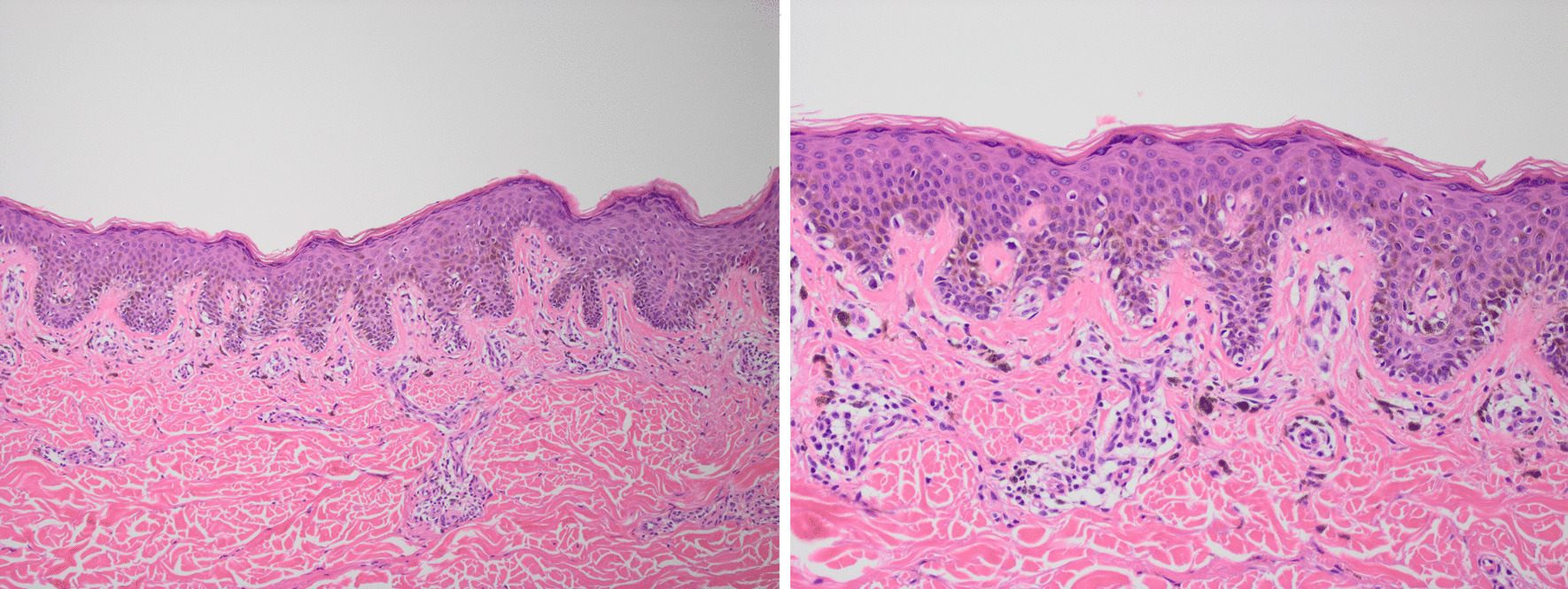
Magnification images, 4× and 10×, of lower back lesion showing markedly increased single and hyperchromatic melanocytes distributed along the base of the epidermis. There is focal upward extension into the epidermis. The pigmented cells seen in the dermis are melanophages

**Fig 3:**
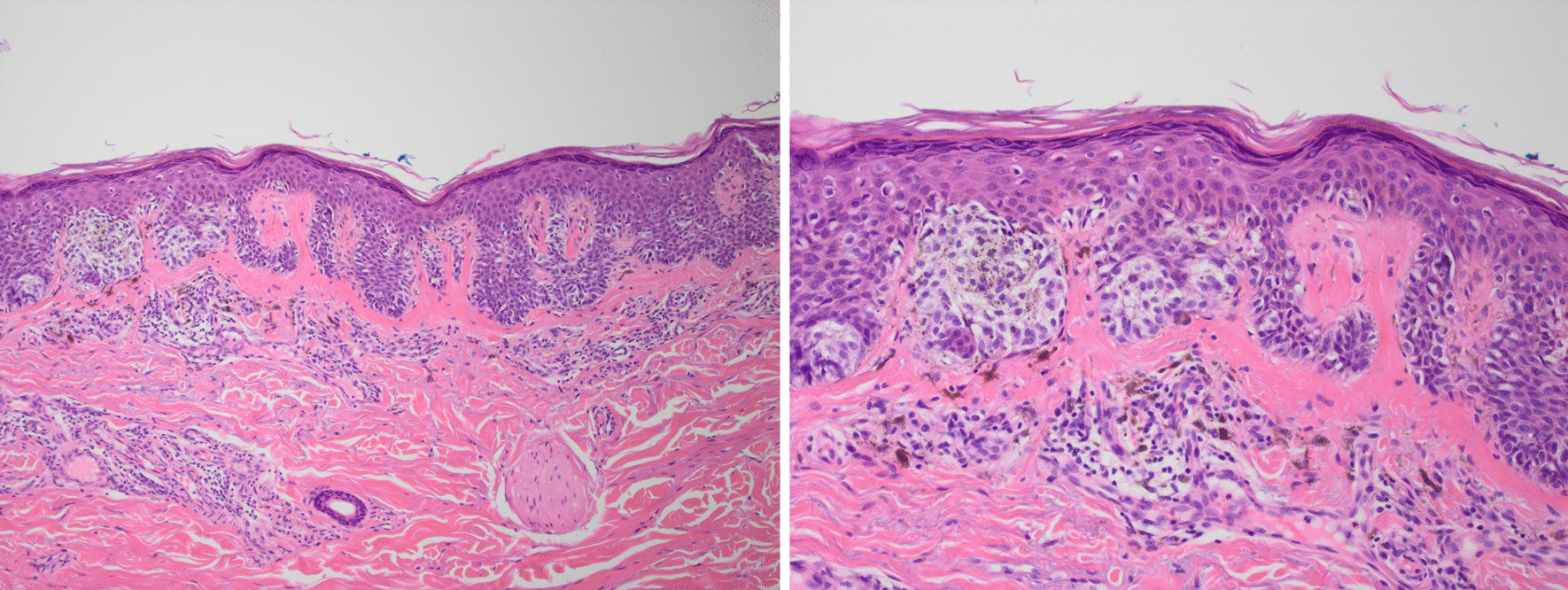
10× and 20× magnification images of the right shoulder lesion. The nests of malignant melanocytes vary markedly in size. Melanocytes are extending up into the epidermis. There is a lymphocytic infiltrate in the dermis, but no invasion

Histopathology examination of the initial excision biopsies was typical for melanoma *in situ*, with nests of malignant melanocytes that varied markedly in size. There were no distinctive features to suggest infection by human immunodeficiency virus. Patients with HIV sometimes show an excess of eosinophils in their inflammatory reaction, but this was not present in this case (Figs. 2, 3).

## Treatment and outcome

Surgical management (after the initial excisional biopsies) was wide excision of the primary tumor sites to ensure minimum 5 mm margins all around, in keeping with clinical guidelines for the management of malignant melanoma. Informed consent was obtained for the procedures, as well as for publication of his images and history in medical journals.

Following excision of the MIS lesions, the patient is well. He is doing well on the HAART therapy and is being followed up 3-monthly for skin checks.

**Fig. 4:**
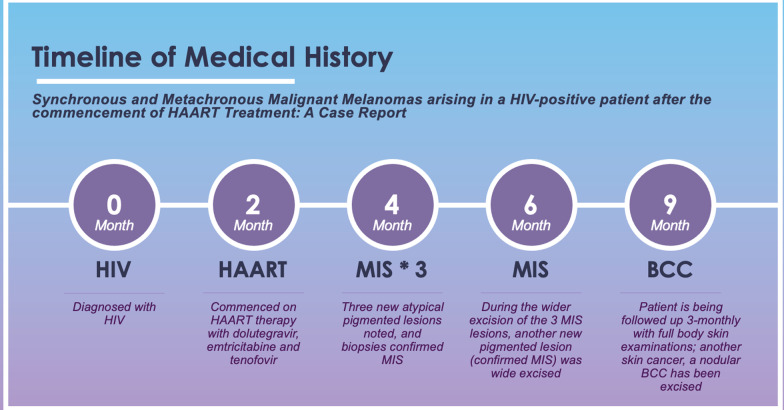
Clinical history timeline

During the 3-monthly full body skin examinations another basal cell carcinoma has since been detected and excised from his trunk. As he is currently 9 months following his HIV diagnosis, we do not have longer-term data. The clinical history timeline is outlined in Fig 4. It is noteworthy that in previous skin examinations prior to his HIV diagnosis, including 2 years previously, he had no suspicious lesions. He also had no previous history of melanoma. He is currently well, asymptomatic both from the point of view of his skin and HIV infection and is being followed up by both skin cancer and infectious disease teams.

## Discussion

A study of the incidence of cutaneous melanoma in HIV-positive patients prior to the introduction of highly active antiretroviral therapy (HAART) in the late 1990s reported an overall standardized incidence ratio for melanoma of 1.24 (95% CI 1.04–1.48) [1]. Pre- and post-HAART studies have not shown any differences in the incidence of melanoma but confirm that people with HIV/AIDS have a 50% increased risk of developing a cutaneous melanoma [2].

Over 70% of primary melanoma patients develop their second melanoma within 5 years of the first malignancy, and over 90% develop their second melanoma within 10 years after the first [3]. While patients with multiple primary invasive melanomas are at increased risk of death compared with patients with single primary invasive melanomas, it is interesting that for patients with melanoma *in situ* (MIS), those with multiple lesions appear to have better outcomes when compared with patients with a single MIS [3]. We are aware of one study that studied the risk of melanoma among individuals infected with HIV by considering only recent CD4 counts that reported elevated relative risks for individuals with CD4 counts under 200 and 201–499 cells/µL, but not for those with CD4 counts greater than 500 cells/µL [4]. A study noted that when compared with uninfected individuals, those with HIV infection and CD4 count < 200 cells/µL had a 44% increased risk of subsequent non-melanoma skin cancer (NMSC) overall and a 222% increase risk of squamous cell carcinoma (SCC) in particular, suggesting that subsequent SCC risk is associated with immune dysfunction, but melanoma was not mentioned similarly [5].

There is a dearth in the literature of evidence regarding the prognosis for patients with multiple primary melanomas compared with those with single primary melanomas [3], and even less in the context of HIV-positive patients. A recent study suggested that overall survival was worse among patients with multiple primary melanomas compared with patients with a single primary melanoma [6]. However, other studies show that, for melanomas in general, prognosis for multiple MIS may indeed be better than for patients with single MIS, and many factors account for this decreased effect size [7, 7].

Longevity for patients with HIV is rising with the advent of HAART [9]. The worldwide incidence of melanoma has also been rising in most fair-skinned populations [10]. Because HAART has turned HIV infection into a chronic disease, patients with advancing age are at a higher risk of cardiovascular, renal, neurological, and, indeed, skin diseases [11]. Therefore, for people living with HIV, immunodeficiency leads to a higher risk of developing malignancies, and is further amplified by risk factors for development of different cancers. In other words, occupational or recreational sun exposure increases the risk of skin cancer even further [12]. Interestingly, there has been a decrease in the incidence of AIDS-defining cancers such as Kaposi’s sarcoma and non-Hodgkin lymphoma, but increases in incidence of many non-AIDS-defining cancers such as skin cancer [13].

Organ transplant recipients treated with immunosuppressive drugs are known to have a higher risk of melanoma, indicating that immunosuppression can trigger melanoma skin cancer [1]. The association between HIV and melanoma is also important to understand better, not only because of the role for immunosuppression in melanoma genesis, but also for the fact that immune checkpoint inhibitors are becoming the mainstay in managing advanced melanoma [14]. We know that tumor-associated antigens are expressed by melanoma cells [15], and most doctors dealing with melanoma would have clinically observed spontaneous tumor regression in some patients with primary melanoma, suggesting some degree of immune modulation [16]. Immunosuppressive drugs also keep organ transplant patients alive for longer, and researchers note that transplant recipients are four times more likely to be diagnosed with regional stage melanoma, that is, melanoma that has already begun to spread to other parts of the body [17]. This finding rules out the possibility that increased skin surveillance of transplant patients is the cause of this increased incidence or diagnosis of melanoma [17].

Melanoma rates are rising globally. New Zealand and Australia—with fair-skinned populations living in environments with high ultraviolet (UV) radiation—have the greatest burden, with disability-adjusted life years scores of 54 [18]. According to the US cancer data supplied by the CDC, from 2013–2017 there were 406,132 new cases of cutaneous melanomas reported, and 43,847 people died of melanomas of the skin, with an overall incidence rate of just under 22 per 100,000 people [19]. CDC figures for HIV infections show that, in 2018, the HIV incidence remained similar to the rate in 2014, with no rise in infection rates. However, while the rate is two-thirds lower than the peak in the 1980s, the rate of new HIV infections has not reduced significantly since 2014 [20]. The estimated number of HIV infections in 2018 was 36,400, and the incidence rate was just over 13 per 100,000 people [20].

The melanoma rate is higher than the rate of HIV infections in countries with fair-skinned populations. However, because melanin is an antioxidant, the degree of immune response (or lack of) that results in a malignant melanoma is worsened by immunosuppression due to drugs (such as used after transplants) or diseases (such as HIV).

## Conclusion

The case presented in this report is unusual because of the rapid development of multiple melanomas over a short period of time in a HIV-positive patient after diagnosis and commencement of treatment. Another reason for this case report is because HIV is not mentioned specifically in melanoma guidelines of care. Fair-skinned people with HIV/AIDS would benefit from close surveillance of their skin for suspicious pigmented lesions because their melanoma rate is increased by 50% [2]. These patients also have a significant, at least twofold, increased risk of developing keratinocyte skin cancers [21] such as basal and squamous cell carcinomas, and therefore should be counseled regarding sun exposure, UV damage, and sun protection.

## Takeaway lessons

- This is an interesting case with multiple melanomas, synchronous and metachronous, developing rapidly in an HIV-positive patient
- While immunosuppression associated with transplant treatment is often associated with higher skin cancer risk, HIV is often overlooked in this regard
- HIV-positive patients with fair skin have a 50% increased rate of developing melanoma and must undergo regular skin surveillance

## Patient perspective

“A few years ago, I had a weird looking mole on my back so went to get a mole check. It was benign. I was not aware that I had any increased risk after my diagnosis as no one told me. I have had mole checks in the past … being a drainlayer and working outside. This time I had myself decided it was time to get my skin checked”

## Data Availability

Not applicable.

## Abbreviations

HAART: Highly active antiretroviral therapy
MIS: Melanoma* in situ*
CDC: Centers for Disease Control and Prevention
BCC: Basal cell carcinoma
SCC: Squamous cell carcinoma
